# Soft frontal swelling in a young girl: Diagnostic nuances and surgical management of a rare case of sinus pericranii

**DOI:** 10.1002/ccr3.9161

**Published:** 2024-07-16

**Authors:** Mobin Ibne Mokbul, Farjana Yesmin, Pukar Gupta, Aminur Rahman, Md. Sumon Rana, Md. Shafiqul Islam

**Affiliations:** ^1^ Dhaka Medical College Dhaka Bangladesh; ^2^ Chitwan Medical College Nepal; ^3^ Department of Neurosurgery Dhaka Medical College Hospital Dhaka Bangladesh

**Keywords:** endovascular embolization, extracalvarial arteriovenous malformation, sinus pericranii, surgery, venous anomalies

## Abstract

A 17‐year‐old girl with a congenital, painless right forehead swelling obstructing her visual field was diagnosed with sinus pericranii. Radiological imaging confirmed extracalvarial arteriovenous malformation with serpentine vessels and bony erosion. Endovascular surgery was chosen for management, highlighting the necessity of considering SP in differential diagnosis for scalp swellings. Despite its rarity, awareness of it is essential to prevent complications from injury, misdiagnosis, or invasive procedures.

## 1 CASE PRESENTATION

**FIGURE 1:**
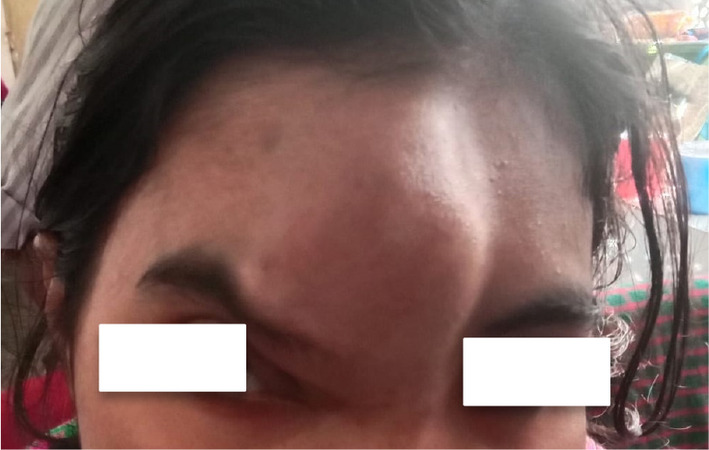
Patient presentation with a frontal bone soft mass of size 7 × 2 cm on the right side.

A 17 year girl presented to our institution loss of right visual field recently. There was a painless, soft tissue swelling which was 7 × 2 cm in dimension in right forehead. (Figure 1) The swelling was there since her birth and had increased in size recently. The mass was immobile, irregular surface, soft in consistency, nontender, nonpulsatile, fluid‐like diffuse swelling with underlying areas of palpable bony defects. The mass did not transilluminate and there was no superficial skin abnormalities. Since the swelling was obscuring her visual field, she experienced difficulty in vision and there was visual difficulty in her right eye recently due to recent increase in size. She also complained of headaches occasionally. On further evaluation, She denied any history of meningitis, tuberculosis, metabolic disorders, jaundice, malaria, or any central nervous system infections in the past. The vision impairment and headache history influenced the decision for further investigations.

Based on the patient's history and physical examination, which one of the following is the most suspicious diagnosis?

- Lipoma
- Arteriovenous malformation (AVM)
- Hemangioma
- Osteomyelitis
- Post‐traumatic cephalocele
- Sinus pericranii

## 2 DIAGNOSIS

**FIGURE 2:**
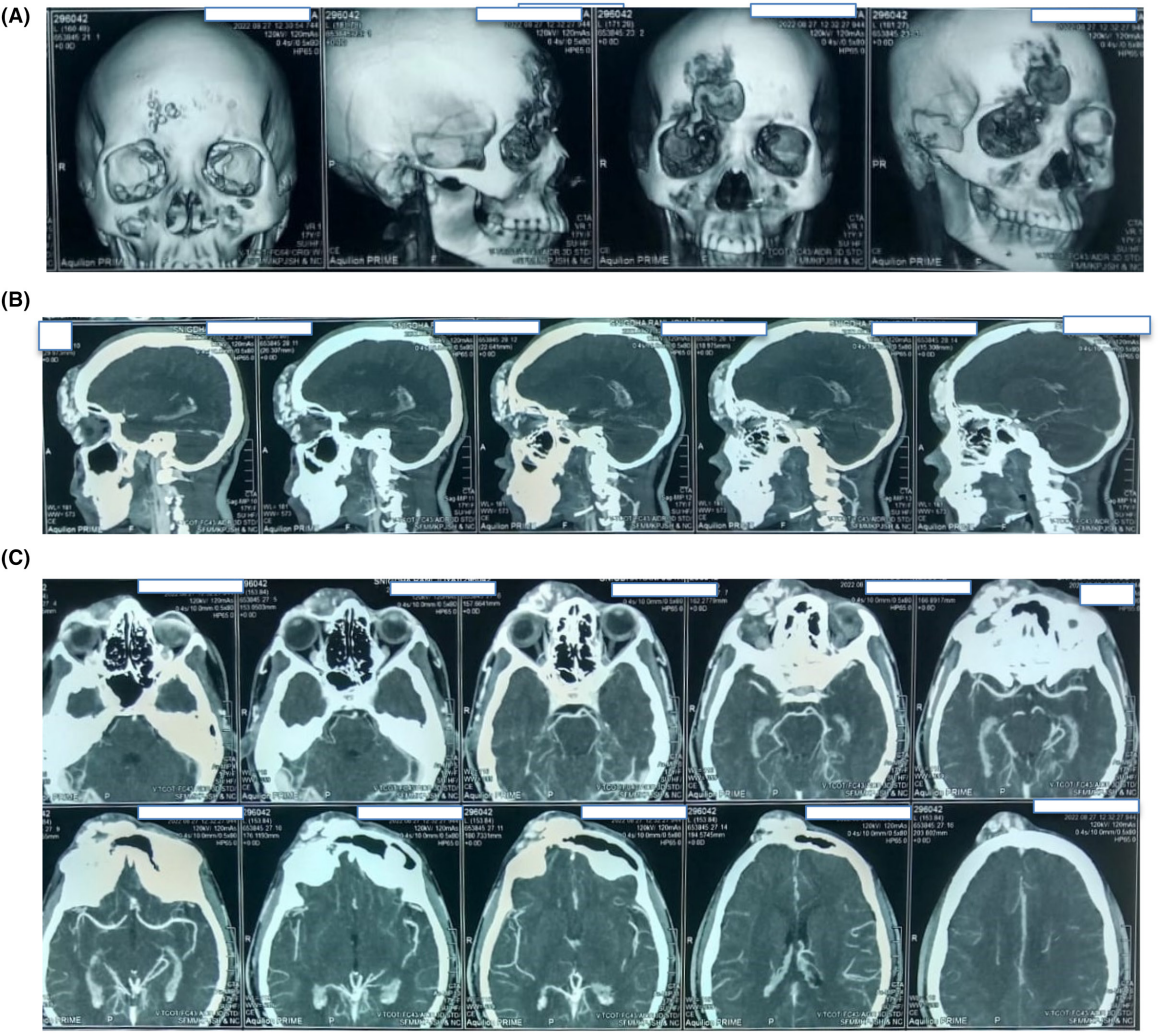
CT Scan of the brain with contrast showing right frontal extracalvarial arteriovenous malformation. (A) Superficial view showing bony defect, (B) Sagittal section, and (C) Transverse section.

The correct answer is (F) Sinus pericranii. CT scan of the brain revealed an extracalvarial irregular serpiginous area of dilated vascular channel (about 6.9 × 2.0) cm at the right frontal region showing serpentine dilated extracalvarial vessels involving the right supraorbital region. (Figure 2) Right ophthalmic and supraorbital vessels were dilated, possibility of supplying the lesion (feeding artery). It had superficial drainage via dilated cortical veins and superior cerebral veins, which drain in the dural venous sinuses and dural frontal‐polar vein were dilated. Erosion of both internal or external plates of calvarium at the frontal region is noted and finally makes the final impression of right frontal extracalvarial arteriovenous malformation as stated above. There was no brain parenchymal enhancement.

**FIGURE 3:**
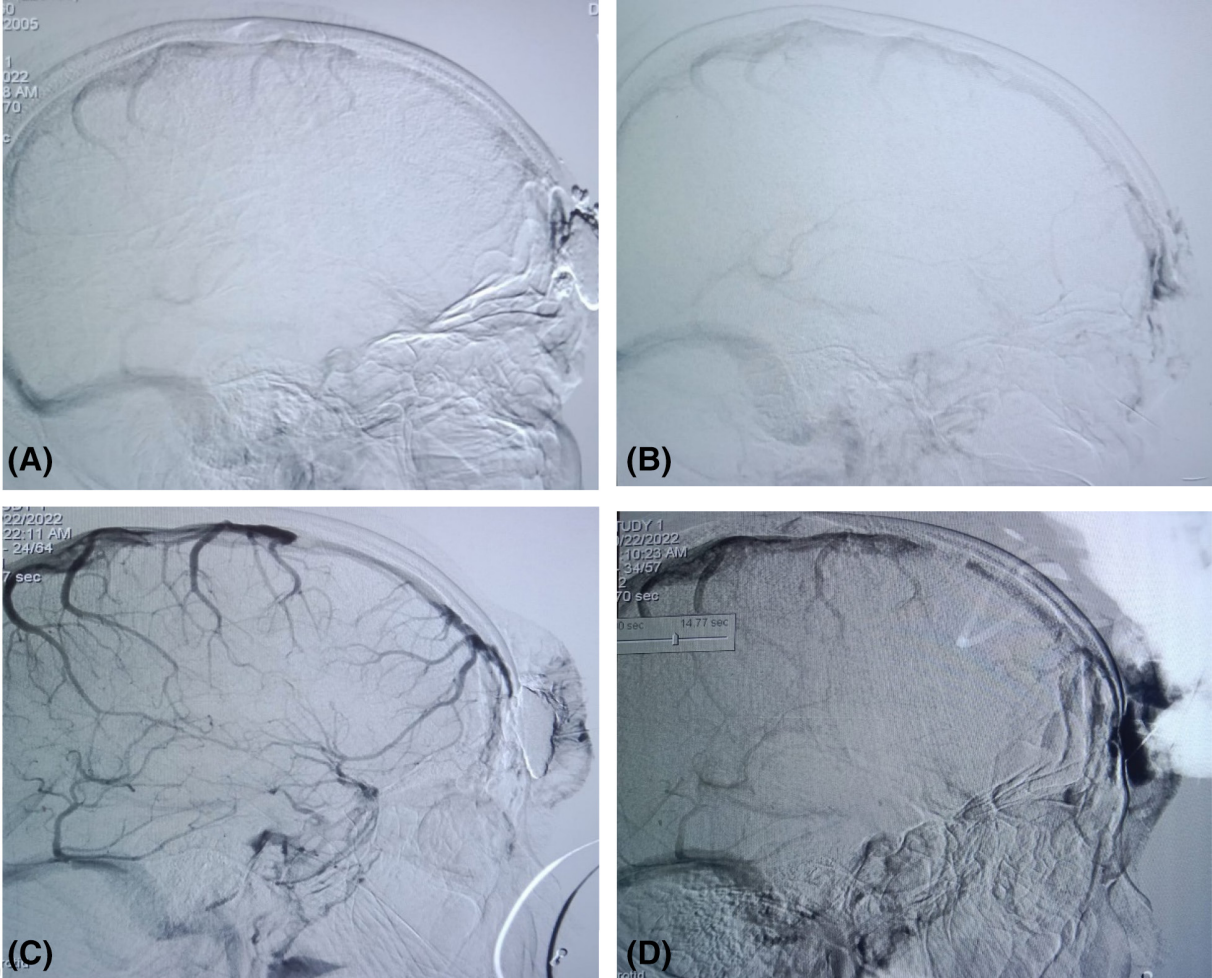
Digital subtraction angiography (DSA) of the extracalvarial malformation. (A, B) Without contrast and (C, D) with contrast.

Digital subtraction angiography (DSA) revealed there was communication between the superior sagittal sinus and supraorbital vein via the emissary vein. (Figure 3) Thus, the diagnosis of accessory sinus pericranii was confirmed.

## 3 OUTCOME

Management guidelines for SP treatment modalities comprise conservative, surgical, and endovascular treatment. Surgical approach is avoided in dominant SP due to significant risk of cerebral bleeding, venous congestion, and cerebral infarction.^1^ In case of accessory SP, open surgery and endovascular embolization can be performed but it is preferred only to prevent future bleeding and thromboembolic events in complex and symptomatic cases and often for cosmetic purpose. We referred her to neuro endovascular surgery department of our hospital for appropriate treatment and she will be undergoing endovascular embolization there. Conservative management was precluded as the size of the lesion was growing and blocking the right visual field. In this case, endovascular embolization was preferred due to its advantages over invasive surgical approach, like short hospital stay, less blood loss, and less infection risks.

## 4 DISCUSSION

**FIGURE 4:**
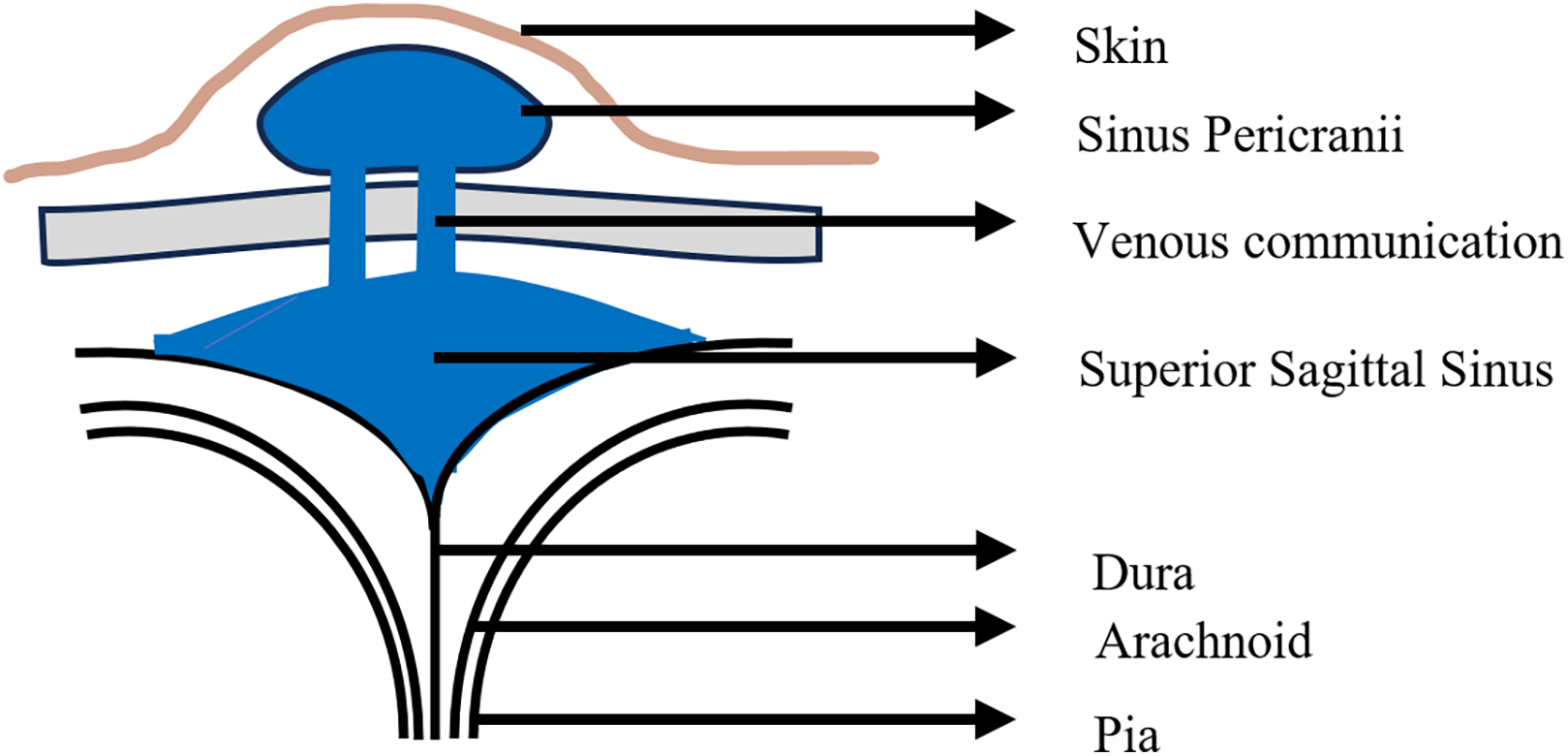
Schematic diagram of sinus pericranii.

Sinus pericranii (SP) is a rare vascular malformation where venous growth connects the scalp to the brain's sinuses.^1^, ^2^, ^3^ Despite being potentially life‐threatening, treatment guidelines are lacking due to limited cases. SP was first described in 1850 by Stromeyer. It is classified by venous flow pattern into dominant (primary brain drainage through SP) and accessory (minor drainage outside the skull).^2^ Figure 4 shows a schematic diagram of the pathology. Our literature search in PubMed revealed only about 220 cases of SP till January 2024. Table 1 illustrates key findings of sinus pericranii from published literature as per our search. A hypothesis suggests that a transient venous hypertension during the final stage of embryogenesis and venous plexus regression between the periosteum and dura mater in the closure of cranial sutures may be responsible for developing SP.^3^ SP should be considered in differential diagnosis of soft extracranial scalp swellings due to its potential for misdiagnosis and significant complications regardless of its rarity. It can be mistaken for more common conditions, leading to inappropriate management. Awareness is crucial as invasive procedures or trauma to the area in cases of SP can result in serious complications like bleeding or venous congestion.

**TABLE 1:** Overview of sinus pericranii.^1^, ^2^, ^3^

Incidence	About 220 cases reported up to 2024 in PubMed
Age	At any age, usually from birth to 30 years
Sex predilection	Slight female predominance in some case series
Etiology	Congenital and trauma
Association	Other vascular, meningocerebral, and systemic anomalies.
Investigation	CDOS, MRI, MRV, CT scan, and DSA (gold standard)
Management	Conservative if asymptomatic Open surgery or Endovascular embolization if symptomatic
Recurrence	Rare
Prognosis	Low risk of spontaneous bleeding Spontaneous involution Partial thrombosis

Abbreviations: CDOS, contrast doppler ultrasonography; CT, computed tomography scan; DSA, digital subtraction angiography; MRI, magnetic resonance imaging; MRV, magnetic resonance venography.

## AUTHOR CONTRIBUTIONS

**Mobin Ibne Mokbul:** Conceptualization; data curation; methodology; supervision; validation; visualization; writing – original draft; writing – review and editing. **Farjana Yesmin:** Visualization; writing – original draft; writing – review and editing. **Pukar Gupta:** Writing – original draft. **Aminur Rahman:** Conceptualization; data curation. **Md. Sumon Rana:** Conceptualization; data curation. **Md. Shafiqul Islam:** Conceptualization; data curation.

## FUNDING INFORMATION

None declared.

## CONSENT

Written informed consent was obtained from the patient to publish this report in accordance with the journal's patient consent policy.

## Data Availability

The data used in this case report is available upon request from the corresponding author.
